# Development and validation of a model that predicts the risk of diabetic kidney disease in type 2 diabetes mellitus patients: a retrospective study

**DOI:** 10.3389/fendo.2025.1708419

**Published:** 2026-01-13

**Authors:** Zhiling Deng, Jian Yang, Hairong Zhou

**Affiliations:** 1 Lihuan Community Health Service Station, The Eighth Affiliated Hospital of Sun Yat-sen University, Shenzhen, China; 2 General Practice Department, The People’s Hospital of Longhua Shenzhen, Shenzhen, China

**Keywords:** diabetic nephropathy, nomogram, prediction model, risk factors, type 2 diabetes mellitus

## Abstract

**Introduction:**

This study aimed to identify independent risk factors for DKD in T2DM patients and develop a risk prediction model with internal validation.

**Methods:**

We retrospectively collected data from 1,049 T2DM patients undergoing community health checks in Longhua District (2024). Patients were divided into DKD and non-DKD groups, then randomly divided into training (n=735) and validation (n=314) sets in 7:3 ratio.

**Results:**

The results of the binary logistic regression analysis showed that the duration of diabetes (OR 1.037, 95% CI: 1.005-1.07, P = 0.024), BMI (OR 0.869, 95% CI: 0.762-0.992, P = 0.037), Scr (OR 1.019, 95% CI: 1.010-1.028, P = 0.000), WBC (OR 1.141, 95% CI: 1.019-1.279, P = 0.023), and TyG-BMI (OR 1.019, 95% CI: 1.1007-1.030, P = 0.002) were independent risk factors for the occurrence of DKD in T2DM. Seven predictors including duration of diabetes, BMI, Scr, WBC, TyG-BMI, hypertension, and HDL-C, which were identified via binary logistic analysis. We visualized the predictive model in the form of a nomogram and evaluated its predictive performance. The model demonstrated good discrimination (AUC: training 0.725, validation 0.698) and calibration (H-L test P>0.05 for both groups). Decision curve analysis confirmed its clinical utility by showing higher net benefit than extreme scenarios.

**Conclusion:**

All seven indicators in this model are readily obtainable in primary healthcare settings, providing a practical tool for primary care physicians to conduct DKD risk prediction in general practice.

## Introduction

With the acceleration of global economic development and the aging of the population, the global prevalence of diabetes (Diabetes mellitus, DM) is also on the rise (1). According to the data projected by the International Diabetes Federation (IDF) in 2021, the incidence rate of DM among adults worldwide is 10.5%. Currently, the number of diabetes patients is as high as 537 million. Moreover, this figure is still on the rise. It is projected that the number of diabetes patients to 643 million in 2045.

T2DM is a chronic and progressive metabolic disease. As the disease progresses, it can cause damage to blood vessels or nerves, resulting in diabetic retinopathy, diabetic nephropathy, diabetic peripheral neuropathy, etc. (2). Diabetic kidney disease (DKD) is one of the common complications of T2DM. Data has shown that, the prevalence of DN in developed countries is 20% to 40% (3). In recent decades, the prevalence of DKD in China has shown a rapid upward trend, which has triggered extremely serious social and economic problems as well as public health crises (4). Approximately one-third of diabetes patients have diabetic nephropathy, and 10% of these patients die from renal failure in China (4). Due to the fact that DKD rarely has symptoms in the early stage, it is difficult to be diagnosed in this stage. When diagnosed, most patients have already reached the middle or late stage of the disease, and the kidneys have suffered irreversible damage. Even quickly progress to end-stage renal disease (ESRD), which requires long-term dialysis or kidney transplantation (5, 6). DKD not only has a negative impact on the quality of life of patients, but also increases the financial burden on local governments. Implementing risk prediction for DKD in patients with T2DM is of great significance in reducing the incidence of severe kidney diseases in type 2 diabetes patients, improving their clinical prognosis, and saving medical costs.

Current studies have confirmed that serum creatinine (Scr), urine albumin-to-creatinine ratio (UACR), and diabetic retinopathy (DR) are closely related to the occurrence of DKD (7, 8). However, the abnormality of these indicators indicates that the patient has already developed DKD, which has certain limitations for the prevention and treatment of the disease. Aiming to solve this problem, researchers have constructed DKD risk prediction models to predict the disease risk of patients with type 2 diabetes. Disease prediction model can to help clinicist to make early diagnosis and treatments, control the risk factors of DKD at the early stage (9, 10).

As a predictive model, the nomogram has been extensively studied and confirmed in the medical field, demonstrating its potential to serve as an effective tool for the initial diagnosis and prognosis assessment of diseases (11, 12). There have been studies developing nomogram prediction models for predicting DKD. However, there are few studies on the development of DKD prediction models in primary healthcare. Based on this, this study focuses on the group of type 2 diabetes patients who undergo physical examinations in primary medical institutions, and conducts a systematic analysis of their relevant clinical data and laboratory indicators. In this study, we analyzed the independent risk factors for type 2 diabetes and constructed a nomogram prediction model based on this, and conducted internal validation. The innovation of this study lies in the fact that the predictive model we constructed includes the inflammatory marker WBC and the insulin resistance index TyG-BMI, both of which can be easily obtained in primary healthcare settings. The significance of this study lies in building a DKD risk prediction model with good predictive performance that is applicable to primary health care, and providing assistance for primary clinical work.

## Methods

### Study design and participations

#### Sample size calculation

The sample size calculation was based on the Events Per Variable (EPV) method. We planned to incorporate approximately 15 candidate variables into a multivariate logistic regression model. With an EPV set at 10, an expected positive event rate of 20%, and a loss to follow-up rate of 10%, the calculated minimum required sample size was 834.

We retrospectively collect clinical data and laboratory test results of T2DM patients who completed physical examinations at the Community Health Service Center of Longhua District People’s Hospital from January 1, 2024 to December 31, 2024. A total of 1,049 people were included in the study.

### Participant grouping

The patients were grouped up into the DKD group and the non-DKD group according to the Guidelines for the Prevention and Treatment of Diabetes and Kidney Disease in China (2021 Edition) (13). Patients who meet one of the following criteria are included in the DKD group: at least 2 out of 3 tests within 3–6 months show a urinary albumin-to-creatinine ratio (UACR) > 30 mg/g or a 24-hour urine albumin excretion rate (UAER) > 30 mg/24 h (20μg/min); or the estimated glomerular filtration rate (eGFR) remains below 60 ml/(min/1.73 m²) for more than 3 months; or renal biopsy reveals pathological changes consistent with DKD. Those who do not meet these criteria are included in the non-DKD group. The eGFR is calculated using the modified Chinese CKD- EPI formula.

### Inclusion criteria and exclusion criteria

Inclusion criteria: (1) Age ≥ 18 years; (2) Diagnosed with T2DM. Exclusion criteria: (1) Presenting with acute complications such as severe infection, diabetic ketoacidosis, hyperosmolar hyperglycemic syndrome, etc.; (2) Suffering from severe dysfunction of the heart or liver organs; (3) Having serious diseases such as malignant tumors, autoimmune diseases, hematological diseases, hyperthyroidism, etc.; (4) Data incomplete, unable to obtain complete demographic, clinical or laboratory test data.

### Data collection

Collect information on gender, age, smoking history, drinking history, duration of T2DM, and calculate the body mass index (BMI) based on the patient’s height and weight. At the same time, collect the patient’s previous medical history of DR, hypertension, stroke, and coronary heart disease(CHD), as well as the usage of three types of medications, including whether ACEI/ARB, SGLT_2 inhibitors, and lipid-lowering drugs were used or not.

Twenty laboratory indicators were included as research variables. These include fasting blood glucose (FBG), glycated hemoglobin (HbA1c), red blood cell count (RBC), white blood cell count (WBC), hemoglobin (HGB), neutrophil count, lymphocyte count, monocyte count, platelet count, mean platelet volume (MPV), aspartate aminotransferase (AST), alanine aminotransferase (ALT), total bilirubin (TBIL), total cholesterol (TC), triglycerides (TG), low-density lipoprotein cholesterol (LDL-C), high-density lipoprotein cholesterol (HDL-C), serum creatinine (Scr), blood urea nitrogen (BUN), and uric acid (UA).

### Definitions

T2DM is diagnosed according to the ADA 2019 Standards of Medical Care in Diabetes (14). Typical symptoms of diabetes (polydipsia, polyuria, polyphagia, weight loss)+random venous plasma blood glucose ≥ 11.1 mmol/L or fasting blood glucose ≥ 7.0 mmol/L or OGTT 2-hour glucose in venous plasma (for those without diabetic symptoms, repeat the test on another day) ≥ 11.1 mmol/L.

The diagnosis of DR was established upon the identification of any of the following pathological alterations: anomalies within the intra-retinal microvascular network, microaneurysms, cotton wool spots, hard exudates, retinal hemorrhages, venous beading (tortuosity), or neovascularization.

Hypertension was delineated as a medical condition wherein the systolic blood pressure (SBP) reached or exceeded 140mmHg, and/or the diastolic blood pressure(DBP) attained or surpassed 90mmHg.

CHD is diagnosed as atherosclerotic coronary artery disease by a tertiary hospital. Stroke is diagnosed by a tertiary hospital as a stroke, or the patient has had a cerebrovascular event in the past and has an imaging report to support it. Individuals who smoke more than 1 cigarette per day for a continuous period exceeding 1 year are defined as smokers. Individuals who consume alcohol at least once a week and average two or more drinking sessions per month are defined as drinkers. The definition of “duration” refers to the time span from the patient’s diagnosis of type 2 diabetes to the date of blood collection.

The formula for calculating BMI is weight (kg)/height (m)². The Neutrophil-to-Lymphocyte Ratio (NLR) is the ratio of neutrophil count to lymphocyte count. The Lymphocyte-to-Monocyte Ratio (LMR) is the ratio of lymphocyte count to monocyte count. The Platelet-to-Lymphocyte Ratio (PLR) refers to the ratio of platelet count to lymphocyte count. The Systemic immune inflammation index (SII) is obtained by multiplying platelet count by neutrophil count and dividing by lymphocyte count (15). The Triglyceride-glucose index (TyG) is calculated from triglyceride value and fasting blood glucose (16). TyG index=Ln[(fasting triglyceride (mg/dL))×fasting blood glucose (mg/dL)/2. The Triglyceride glucose body mass index (TyG-BMI) formula is TyG index×BMI value.

### Ethical review

This study has been reviewed and approved by the Ethics Committee of Longhua District People’s Hospital, which granted permission to conduct this retrospective study (Ethics Approval Number: Longhua Renyi Lunshen (Research) [2025] No. (087)). Given that the present study exclusively employs anonymized historical clinical data, entails no intervention procedures whatsoever, and presents no more than minimal risk to the research participants, the Ethics Committee has granted an exemption from the requirement to obtain informed consent from the patients.

### Statistical analysis

The data processing software used included IBM SPSS 27.0 and R software (version 4.2.0), with a significance level of P = 0.05. For measurement data adhering to a normal distribution, the mean and standard deviation (x ± s) are utilized for descriptive statistics, and the t-test is employed to assess intergroup differences. Conversely, for data not conforming to a normal distribution, the median and interquartile range [M(P25, P75)] are used to characterize the data distribution, with the Mann-Whitney U rank sum test applied for group comparisons. Qualitative data are represented by the number of cases and corresponding percentages (%). When evaluating differences between groups of categorical variables, either the χ2 test or the rank sum test is appropriate. The data were randomly split into a training set and a validation set in a 7:3 ratio using a random function.

Univariate logistic regression analysis was used to analyze the variables. The variables with statistical significance were included in the binary Logistic multivariate regression to screen out the independent risk factors for DKD. Backward stepwise iterative modeling is one of the methods for constructing Logistic regression models. In this study, the presence or absence of DKD was taken as the dependent variable. Variables with statistical differences, screened through univariate analysis, were incorporated into a binary Logistic multivariate regression analysis framework. The model underwent stepwise iteration to identify the final predictive factors, and a Logistic regression equation was subsequently constructed based on the regression coefficients of these predictors.

Using the rms package in the statistical software R, convert regression coefficients into scale marks and generate visual graphics, namely nomograms. The Hosmer-Lemeshow (H-L) test was employed to evaluate the goodness of fit of the model. The discriminatory ability of the model was evaluated using the receiver operating characteristic (ROC) curve and the area under the ROC curve (AUC). The clinical practicability of the prediction model is evaluated using the Clinical Decision Curve Analysis (Decision Curve Analysis, DCA).

## Result

### Baseline characteristics of participants

This study included a total of 1049 patients with type 2 diabetes mellitus. Using the random number table method, all the subjects were randomly divided into a training set of 735 patients and a validation set of 314 patients in a 7:3 ratio. According to the diagnostic criteria, the patients in the training set were divided into the DKD group and the non-DKD group. Statistical analysis was conducted on the data of the training group, DKD group and non-DKD group. There were statistically significant differences among the groups in terms of gender, combined hypertension, smoking, use of ACEI or ARB drugs, BMI, FGB, TG, TC, BUN, SCr, UA, HbA1c, WBC, RBC, ALC, MPV, NLR, Plt-SII, TyG, and TyG-BMI (P < 0.05), as shown in **Supplementary Table 1**.

### Univariate and multivariate analyses of risk predictors

**Supplementary Table 2** lists the variables identified as predictors of DKD incidence in patients with T2DM. Univariate logistic regression analysis showed that there were statistically significant differences in TyG-BMI, TyG, HGB, AMC, ANC, WBC, HbA1c, hypertension, smoking, FBG, BMI, HDL-C, TG, UA, SCr, BUN, duration of diabetes, and the use of ACEI/ARB (P < 0.05).

Based on the results of univariate logistics, the risk factors related to the risk of DN in T2DM patients were further screened by backward stepwise regression. The results showed that the duration of diabetes (OR 1.037, 95% CI: 1.005-1.07, P = 0.024), BMI (OR 0.869, 95% CI: 0.762-0.992, P = 0.037), Scr (OR 1.019, 95% CI: 1.01-1.028, P = 0.000), WBC (OR 1.141, 95% CI: 1.019-1.279, P = 0.023), and TyG-BMI (OR 1.019, 95% CI: 1.1007-1.03, P = 0.002) were independent risk factors for the occurrence of DKD in T2DM, as shown in **Supplementary Table 3**. It is noteworthy that in this study, variables found to have significant correlations in the univariate analysis, namely hypertension (OR 1.785, 95% CI: 1.227-2.596, P = 0.002) and HDL-C (OR 0.223, 95% CI: 0.109-0.455, P = 0.000), no longer showed significant correlations with the occurrence of DKD in the multivariate regression analysis (P > 0.05), with hypertension (OR 1.46, 95% CI: 0.975-2.187, P = 0.067) and HDL-C (OR 0.563, 95% CI: 0.260-1.220, P = 0.145). This is because collinearity may exist among variables in binary logistic regression analysis.

### Construction of the nomogram prediction model

We constructed a predictive model for DKD that incorporates seven predictors screened by binary logistic regression: duration of diabetes, BMI, Scr, WBC, TyG-BMI, hypertension, and HDL-C. According to the regression coefficients of each independent variable obtained from the logistic regression analysis, the constructed logistic regression equation is as follows: Logit(P) = -4.224 + 1.037×Duration of DM + 1.46×Hypertension (= 0 or 1) +0.869×BMI + 0.563×HDL-C+1.019×Scr + 1.141 WBC + 1.019×TyG-BMI. Visualize the predictors screened by logistic regression in the form of a nomogram, shown in **Supplementary Figure 1**.

The risk value corresponding to the total score in the nomogram represents the probability of patients developing DKD in the context of T2DM. The total score is obtained by adding up the scores corresponding to each prediction indicator. As an example to better explain the nomogram, if the patient has a diabetes duration of 7 years, without suffering from hypertension, has a BMI of 25.28 kg/m^2^, an HDL-C level of 1.49 mmol/L, a Scr value of 82.6 µmol/L, a white blood cell count of 10.26×10^9^/L, and TyG-BMI index is 209.78, the patient’s score on the nomogram is -4.06 points, and the probability of DN was estimated to beis 11.3% (**Supplementary Figure 2**).

### Evaluation and validation of the prediction model

The AUC of the training set was 0.725 (95% CI: 0.68-0.769), When distinguishing between high and low risk using the optimal predictive risk cutoff value of 0.147, the specificity was 0.537 and the sensitivity was 0.813, indicating good discrimination (**Supplementary Figure 1A**). In the validation group, the AUC was 0.698 (95% CI: 0.627-0.770). Using 0.194 as the cutoff for high and low risk stratification, the specificity was 0.697 and the sensitivity 0.635 (**Supplementary Figure 1B**). The H-L test showed P = 0.878 in the training set (**Supplementary Figure 2A**) and P = 0.850 in the validation set (**Supplementary Figure 2A**), showing good consistency between the predicted and ideal curves (P > 0.05).

### Clinical use

The decision curve of the DKD risk nomogram is shown in **Supplementary Figure 3**. According to decision curve analysis, nomograms are clinically beneficial in predicting the risk of DKD incidence between a considerable range of threshold probabilities (15%-90%).

## Discussion

DKD is a common complication of type 2 diabetes. Due to the fact that symptoms are rarely present in the early stage of the disease and it is often diagnosed only in the later stages, kidney function cannot be reversed at that stage, which is very unfavorable for the prognosis of DKD (17). Therefore, establishing predictive models for early diagnosis is of utmost importance. Disease risk prediction models are clinical decision tools that can help clinicians accurately predict the occurrence of the disease. Although the number of studies on DKD prediction models in China had significantly increased over the past five years, only a small proportion of these studies were carried out in primary healthcare settings (18).

In this study, we established a model for predicting DKD in D2TM patients in primary healthcare settings. It included seven predictors: duration of diabetes, hypertension, BMI, HDL-C, WBC, Scr, and TyG-BMI. Both the AUC and DCA of the model confirm its excellent predictive performance. All these indicators can be obtained in primary healthcare settings, which makes the widespread application of the model possible.

The prevalence of DKD in our study was 19.26%, which is consistent with the previous literature reports (19). However, globally, the incidence rate of DKD varies significantly. A 15-year observational study from January 1989 to January 2004 in Saudi Arabia showed that the incidence rate of DKD was approximately 32.1% (20). The geographical variations in incidence rates may be related to factors such as the patient visit habits in different regions, the medical level in different regions, and the differences in the medical insurance systems.

Duration of the disease is an independent risk factor for the occurrence of DKD. This viewpoint has already been confirmed in previous studies. As the duration of DM progresses, the number and function of pancreatic islet cells in the body are increasingly impaired. Long-term poor blood sugar control can easily lead to damage of the renal vascular system, thereby accelerating the progression of renal function (21). In 2022, a cross-sectional study in Shanghai involving 883 patients with T2DM (22) results confirmed that with the prolongation of the disease course, the prevalence of DKD in patients with T2DM increased. The data from this study showed that the incidence of DKD in T2DM patients with a disease course of less than 8 years was 28.38%, while it increased to 38.53% for those with a disease course of more than 8 years. A 12-year follow-up study in Pakistan (23) also reached a similar conclusion. The study found that with the prolongation of the T2DM disease course, the incidence of DKD gradually increased, and the renal function of patients showed varying degrees of decline. The research by Jiang W (24) et al. pointed out that approximately 25% of T2DM patients with a disease course of more than 10 years would develop microalbuminuria. Wang J (25) et al. conducted a retrospective analysis of 505 T2DM patients diagnosed by renal biopsy, and the results showed that the duration of diabetes was an independent risk factor for the occurrence and development of DKD. For every additional year of disease course, the risk of DKD increased by 1.015 times. In our study, the duration of illness among patients in the DKD group was 6 (3.41,13.67) years. This serves as a crucial reminder for clinicians that enhanced vigilance and more frequent screening for DKD are warranted in patients with long-standing T2DM.

Hypertension is regarded as a common comorbidity of diabetes. When the body is in a state of persistent elevated blood pressure, the renin-angiotensin-aldosterone system is activated, causing the kidneys to enter a state of high perfusion. The continuous high perfusion state further leads to arteriosclerosis of the renal arteries, resulting in thickening of the vessel walls and narrowing of the lumen, which in turn causes a reduction in renal blood flow, ultimately leading to kidney damage and the occurrence of DKD (26, 27). Jieqiong Lou et al. (28) conducted a study covering 6 communities in Shanghai, involving a total of 5,078 samples, which also confirmed this conclusion. When patients with T2DM have hypertension, the incidence of DKD increased from 53.5% to 59%. Further comparative analysis revealed that compared with T2DM patients without hypertension, the risk of DKD in patients with hypertension was 1.442 times that of those without hypertension. However, in this study, we found that hypertension showing significant correlations in univariate analysis, but no longer demonstrated significant correlations with the occurrence of DKD in multivariate regression analysis. This is because collinearity may exist among variables in binary logistic regression analysis.

The result shows that BMI is negatively correlated with the risk of developing DKD in our study is worthy of further in-depth exploration. Based on the findings of previous studies, BMI is associated with multiple complications of T2DM. The risk of coronary heart disease was significantly reduced, while a BMI ≥ 25 kg/m^2^ increased the risk of heart failure in patients (29). A retrospective study in Guangzhou, China found that a BMI greater than 25.0 kg/m^2^ was a protective factor for treatment-requiring diabetic eye disease (30), with an OR of 0.57 (95% CI 0.33-0.96). Current research has proved that the correlation between BMI and DKD is not a simple linear correlation, but rather presents a U-shaped or L-shaped non-linear correlation. Shi Shaomin et al. (31) conducted a retrospective study in Xiangyang, China. The correlation between BMI, waist-hip circumference ratio (WHR), visceral fat tissue area (VFA) and DKD showed a U-shaped correlation, indicating that within a certain range, BMI has a positive correlation with the occurrence of DKD, while exceeding a certain value, BMI may become a protective factor for the occurrence of DKD. However, the study did not provide the specific numerical value for the turning point of the correlation. In 2025, a study involving approximately 110,000 patients from the national medical claims database in Japan (29) showed that the relationship between the time when T2DM patients began kidney dialysis and BMI, waist circumference (WC) was not linear but L-shaped correlation. When BMI≥25 kg/m^2^ and WC≥90 cm, the risk of dialysis was significantly reduced (HR = 0.42, 95% CI = 0.29-0.62). In other words, among diabetic patients, those with a BMI≥25kg/m^2^ exhibit a decreasing trend in the risk of kidney dialysis. This is consistent with our research findings. As described earlier in our study, the BMI of patients in the DKD (Diabetic Kidney Disease) group was 25.07 (22.99, 28.26) kg/m². However, larger sample size studies are still needed in the future to verify the impact of BMI in different stratifications on the occurrence of DKD.

Our study found that HDL-C is a protective factor while Scr is an independent risk factor for DKD. HDL-C mainly plays the role of reverse cholesterol transport in the body, which can promote the clearance of cholesterol in peripheral tissues and help maintain the balance of lipid metabolism. It is a protective factor for cardiovascular diseases. Numerous studies have confirmed (32, 33) that the level of HDL-C is significantly correlated with the occurrence and progression of diabetic nephropathy, and low levels of HDL-C are an independent predictor of diabetic nephropathy. This is the same as the results of this study. HDL-C was a protective factor for the occurrence of DKD (OR = 0.563, 95% CI: 0.26-1.22, P = 0.145). A retrospective analysis study by Xia et al. (34) discovered that elevated levels of SCr, CysC, TC, TG, LDL-C, and decreased HDL-C were all independent risk factors affecting the occurrence of DKD (P < 0.05). In recent years, studies have combined Scr with other DKD risk factors for detection to predict the risk of DKD occurrence. A study by Zhai JJ (35) on a small sample size retrospective study found that the area under the curve for the combined detection of Scr, fibrinogen, D-dimer, cystatin C, and homocysteine for diagnosing DKD was significantly higher than that of each indicator alone. This indicates that the diagnostic accuracy of the combined detection of these indicators is higher than that of each indicator alone. Similarly, the DKD risk prediction model we developed also employs a combined prediction strategy using Scr, HDL and other indicators, aiming to enhance the clinical predictive capability for the onset of DKD.

White blood cells play a promoting role in the inflammatory response and tissue damage of diabetic nephropathy. In a study by Oguiza Ainhoa et al. (35) on patients with diabetic nephropathy, it was found that the infiltrating white blood cells in the kidneys were associated with increased expression of inflammatory factors, suggesting that white blood cells might exacerbate kidney damage by releasing inflammatory mediators. In this study, the univariate logistic analysis of the training group showed that there were statistically significant differences in AMC, ANC, and WBC between the DKD group and the non-DKD group. The results of the multivariate logistic analysis further indicated that WBC was an independent risk factor for the occurrence of DKD (OR 1.141, 95% CI: 1.019 - 1.279, P = 0.023). Although white blood cell counts are easily obtainable in primary healthcare settings, their application in predicting DKD is quite limited. More extensive and multi-center prospective studies with large sample sizes can be conducted to further explore the correlations among different subgroups of WBC cells, as well as other cells such as renal tubular epithelial cells and mesangial cells.

Compared with the traditional methods for evaluating RI, the TyG index, as an emerging derivative indicator for evaluating RI, has been widely applied in clinical practice in recent years. TyG-BMI, as a combined parameter, can more comprehensively reflect the level of insulin resistance (36). Our research findings show that in patients with T2DM, an increase in TyG-BMI is associated with an elevated risk of developing DKD. The research results by Huang N (37) et al. showed that the TyG-BMI of patients with T2DM and proteinuria was significantly higher than that of patients without proteinuria (232.16 [206.52-268.02] vs 229.83 [206.11-255.64], P = 0.023). It is worth noting that the study further conducted a correlation analysis on the original TyG-BMI indicators, and the results showed that the TyG-BMI of T2DM patients was significantly positively correlated with BMI (r=0.866, P< 0.001), TG (r=0.630, P< 0.001), TC (r=0.119,P < 0.001), HDL-C (r=-0.374,P < 0.001), FBG (r=0.297, P< 0.001), and HbA1c (r=0.116, P < 0.001). This reveals that the promoting effect of TyG-BMI on DKD is multifaceted, involving factors such as lipid disorder, poor blood sugar control, and overweight. The TyG-BMI index, which is increasingly found to be associated with chronic diseases, is calculated based on triglyceride levels, fasting blood glucose, and BMI, and can be easily obtained in primary healthcare settings. Our study may enhance the attention and clinical utilization of TyG-BMI among primary care physicians.

### Limitations

Despite its strengths, our study has certain limitations. Firstly, this study adopts a retrospective research design, which inevitably leads to recall bias and case selection bias. Secondly, the diagnosis of DKD was made based on the diagnostic criteria through clinical examination, and no renal biopsy was performed. Thirdly, the clinical data included in this study all come from a single medical center, and the sample size is relatively limited, lacking external data validation. This may to some extent affect the stability and universality of the prediction model. Therefore, in the future, more in-depth exploration of the potential risk factors for DKD will be carried out through multi-center prospective studies.

## Conclusion

Our research results show that the longer the duration of diabetes, the lower BMI, the higher level of Scr, the higher level of WBC and the higher level of TyG-BMI are independent risk factors for the occurrence of DKD in T2DM. The seven indicators in our model include data from medical history collection, physical examination, and laboratory tests, all of which are readily obtainable in primary healthcare settings. This model demonstrates high discrimination and calibration, has good predictive performance, and can accurately analyze the risk of DKD occurrence, thereby providing an effective screening tool for the early identification of high-risk individuals for DKD in primary healthcare settings.

## Data Availability

The raw data supporting the conclusions of this article will be made available by the authors, without undue reservation.
