# Supplementary material for: Development and validation of a model that predicts the risk of diabetic kidney disease in type 2 diabetes mellitus patients: a retrospective study
Source: Front Endocrinol (Lausanne). 2026 Jan 13;16:1708419. doi: 10.3389/fendo.2025.1708419 (PMC12834776; doi:10.3389/fendo.2025.1708419)
Supplement: Supplementary file 1 [file DataSheet1.pdf]

Supplementary

Table 1 Comparison of data between the N-DKD group and the DKD group in the training set

variabl	N-DKD O-group（n=596）	DKD group（n=139）	*χ2/t/Z*	*P*
Gender			3.501	0.061
Female	262(44.0)	49(35.3)		
Male	334(56.0)	90(64.7)		
DR(n,%)			0.035	0.852
No	497(83.4)	115(82.7)		
Yes	99(16.6)	24(17)		
Hypertension(n,%)			9.326	0.002
No	330(55.4)	57(41)		
Yes	266(44.6)	82(59)		
Cardiovascular Disease(n,%)			0.000	0.985
No	532(89.3)	124(89.2)		
Yes	64(10.7)	15(10.8)		
Stroke(n,%)			0.000	0.857
No	564(94.6)	131(94.2)		
Yes	32(5.4)	8(5.8)		
Smoke(n,%)			7.958	0.005
Never	421(70.6)	81(58.3)		
Ever/Current	175(29.4)	58(41.7)		
Alcohol(n,%)			3.311	0.069
Never	448(75.2)	94(67.6)		
Ever/Current	148(24.8)	45(32.4)		
ACEI/ARB drug use(n,%)			4.828	0.028
No	445(74.7)	91(65.5)		
Yes	151(25.3)	48(34.5)		
Lipid-lowering drug use(n,%)			0.048	0.827
No	430(72.1)	99(71.2)		
Yes	166(27.9)	40(28.8)		
SGLT_2_Inhibitor drug use(n,%)			1.722	0.189
No	387(64.9)	82(59)		
Yes	209(35.1)	57(41)		
Age(years)	58(52.65)	58(52,69)	-1.458	0.145
BMI(kg/m2)	24.57(22.8,26.77)	25.07(22.99,28.26)	-2.041	0.041
Diabetes duration(years)	6(3,10)	6(3.41,13.67)	-1.57	0.117
FBG(mmol/L)	7.06(6.12,8.12)	7.5(6.54,8.89)	-3.107	0.002
TBIL(μmol/L)	12.05(9.65,15.3)	11.8(9.6,15.4)	-0.036	0.972
ALT(U/L)	20.65(15,28)	22(15,32)	-0.854	0.393
AST(U/L)	20(17,24.7)	20(18,24)	-0.403	0.687
TG(mmol/L)	1.34(0.93,1.9)	1.69(1.22,2.27)	-4.431	0.000
TC(mmol/L)	4.768±1.126	4.756±1.128	-4.515	0.000

**variabl**	**N-DKD O-group（n=596）**	**DKD group（n=139）**	***χ2/t/Z***	***P***
HDL-C(mmol/L)	1.24(1.05,1.46)	1.1(0.98,1.29)	-0.047	0.962
LDL-C(mmol/L)	3.07(2.35,3.75)	3.07(2.41,3.64)	-0.676	0.499
BUN(mmol/L)	5.72(4.87,6.82)	5.62(4.89,7.18)	-4.898	0.000
SCr(μmol/L)	66.25(56.55,80.05)	75.3(64.9,92.1)	-2.884	0.004
UA(μmol/L)	328(276,401)	358(299,429)	-3.484	0.000
HbA1c(%)	6.8(6.17,7.61)	7.2(6.51,7.97)	-4.308	0.000
WBC(10^12/L)	5.92(4.7,7.05)	6.65(5.54,7.88)	-4.107	0.000
RBC(10^12/L)	4.8(4.49,5.15)	4.9(4.51,5.21)	-1.784	0.074
PLT(10^9/L)	220(190,262)	221(184,263)	-0.452	0.651
HGB(g/L)	144(133,154)	146(137,156)	-0.066	0.947
ANC	3.44(2.76,4.23)	4(3.12,4.82)	-1.564	0.118
ALC	1.88(1.56,2.3)	1.95(1.61,2.42)	-2.711	0.007
AMC	0.33(0.26,0.41)	0.35(0.29,0.42)	-1.533	0.125
MPV	9.4(8.7,10)	9.3(8.6,9.9)	-2.083	0.037
NLR	1.81(1.43,2.3)	1.92(1.51,2.56)	-2.004	0.045
LMR	5.92(4.64,7.53)	5.48(4.44,6.83)	-1.665	0.096
PLR	116.72(95.4,142.64)	108.6(94.01,137.02)	-1.743	0.081
Plt-SII	400.95(292.75,546.6)	424.3(333.64,582.15)	-4.899	0.000
TyG	8.94(8.5,9.37)	9.24(8.85,9.66)	-4.899	0.000
TyG-BMI	219.63(197.79,246.6)	230.21(208.17,269.65)	-3.611	0.000

Table 2 Univariable Logistic Analysis to Extract the Potential Predictors.

**Varibale**	β	S.E.	Z	P value	OR	(95%CI)
TyG-BMI	0.011	0.002	20.181	0.000*	1.011	1.006～1.016
TyG	0.598	0.13	21.038	0.000*	1.818	1.408～2.347
HGB	0.013	0.006	4.281	0.039*	1.013	1.001～1.025
AMC	1.912	0.684	7.814	0.005*	6.768	1.771～25.865
ANC	0.267	0.069	14.755	0.000*	1.306	1.140～1.496
WBC	0.226	0.054	17.425	0.000*	1.254	1.127～1.394
HbA1c	0.168	0.059	8.234	0.004*	1.183	1.055～1.328
Hypertension	0.579	0.191	9.186	0.002*	1.785	1.227～2.596
Smoke	0.544	0.194	7.85	0.005*	1.723	1.178～2.52
FBG	0.099	0.036	7.423	0.006	1.105	1.028～1.186
BMI	0.083	0.029	8.241	0.004*	1.087	1.027～1.151
HDL-C	-1.501	0.364	17.015	0.000*	0.223	0.109～0.455
TG	0.147	0.049	8.875	0.003*	1.159	1.052～1.277
UA	0.003	0.001	8.322	0.004*	1.003	1.001～1.005
SCr	0.023	0.004	28.162	0.000*	1.023	1.014～1.031
BUN	0.119	0.057	4.371	0.037*	1.126	1.007～1.258

**Varibale**	β	S.E.	Z	P value	OR	（95%CI）
Duration	0.034	0.015	5.278	0.022*	1.034	1.005～1.064
ACEI/ARB	0.441	0.202	4.782	0.029*	1.554	1.047～2.308

S.E., Standard error; P＜0.05, with statistical difference.

Table 3 Multivariate Logistic Regression Analysis of Training Set.

**Varibale**	**β**	**S.E.**	**Z**	**P value**	**OR**	**（95%CI）**
Diabetes duration	0.036	0.016	5.069	0.024	1.037	1.005～1.07
Hypertension	0.378	0.206	3.367	0.067	1.46	0.975～2.187
BMI	-0.14	0.067	4.331	0.037	0.869	0.762～0.992
HDL-C	-0.575	0.395	2.122	0.145	0.563	0.26～1.22
SCr	0.019	0.004	17.302	0.000	1.019	1.01～1.028
WBC	0.132	0.058	5.203	0.023	1.141	1.019～1.279
TyG-BMI	0.018	0.006	9.946	0.002	1.019	1.007～1.03
