# Supplementary material for: Development and validation of a model that predicts the risk of diabetic kidney disease in type 2 diabetes mellitus patients: a retrospective study
Source: Front Endocrinol (Lausanne). 2026 Jan 13;16:1708419. doi: 10.3389/fendo.2025.1708419 (PMC12834776; doi:10.3389/fendo.2025.1708419)
Supplement: Supplementary file 2 [file Image1.pdf]

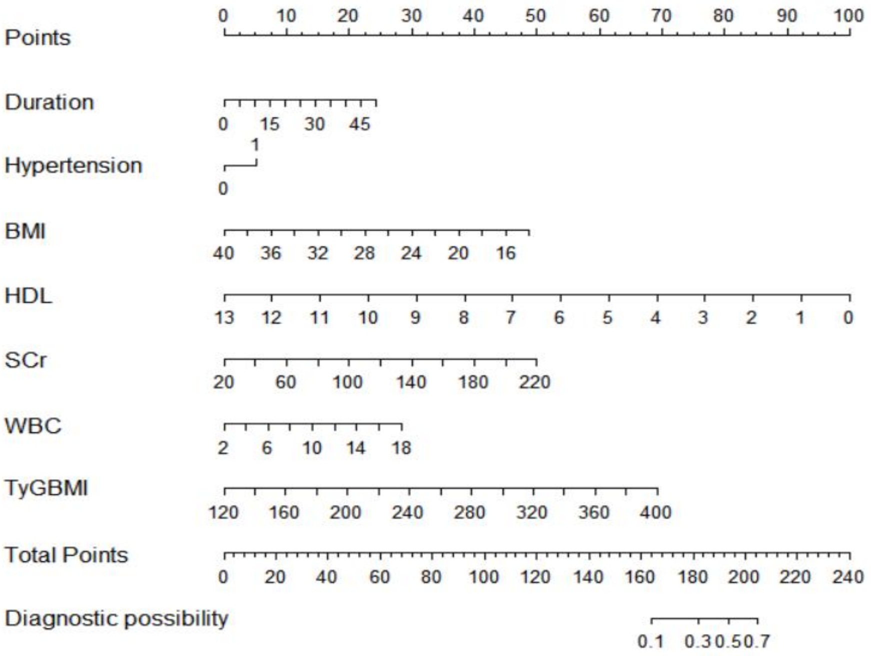

FIGURE 1 Nomogram to predict the risk of DKD for T2DM.

BMI,body mass index; HDL,high-density lipoprotein cholesterol; Scr,serum creatinine; WBC,white blood cell; TyG-BMI,triglyceride glucose body mass index.
