# Supplementary material for: Development and validation of a model that predicts the risk of diabetic kidney disease in type 2 diabetes mellitus patients: a retrospective study
Source: Front Endocrinol (Lausanne). 2026 Jan 13;16:1708419. doi: 10.3389/fendo.2025.1708419 (PMC12834776; doi:10.3389/fendo.2025.1708419)
Supplement: Supplementary file 3 [file Image2.pdf]

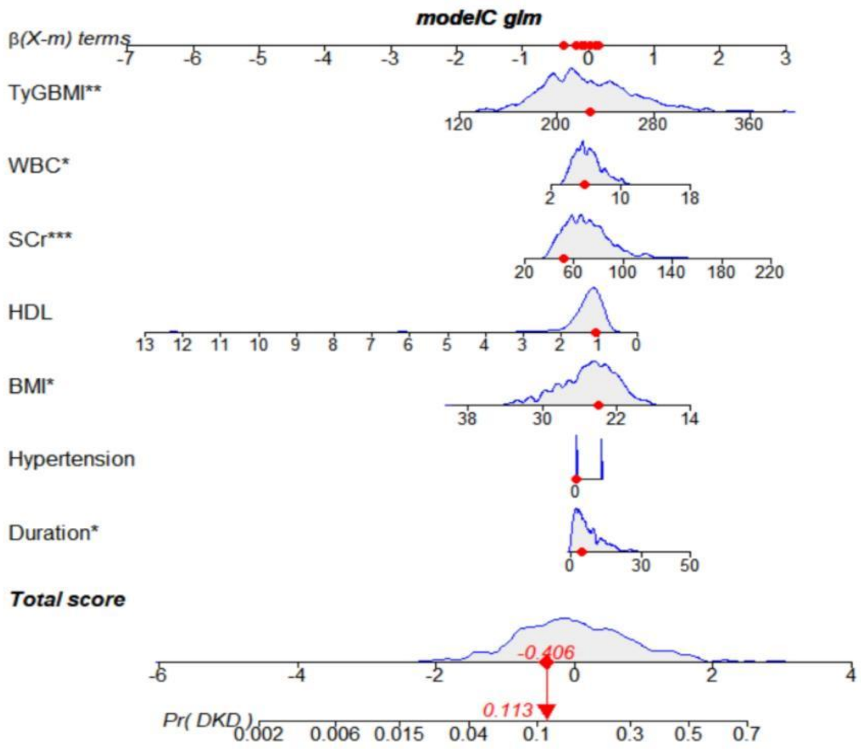

FIGURE 2 An example of nomogram for DKD.

If the patient has a diabetes duration of 7 years, without suffering from hypertension, has a BMI of 25.28 kg/m2, an HDL-C level of 1.49 mmol/L, a Scr value of 82.6 μmol/L, a white blood cell count of 10.26×109/L, and TyG-BMI index is 209.78, the patient's score on the nomogram is -4.06 points, and the probability of DN was estimated to beis 11.3%.
