# Supplementary material for: Development and validation of a model that predicts the risk of diabetic kidney disease in type 2 diabetes mellitus patients: a retrospective study
Source: Front Endocrinol (Lausanne). 2026 Jan 13;16:1708419. doi: 10.3389/fendo.2025.1708419 (PMC12834776; doi:10.3389/fendo.2025.1708419)
Supplement: Supplementary file 4 [file Image3.pdf]

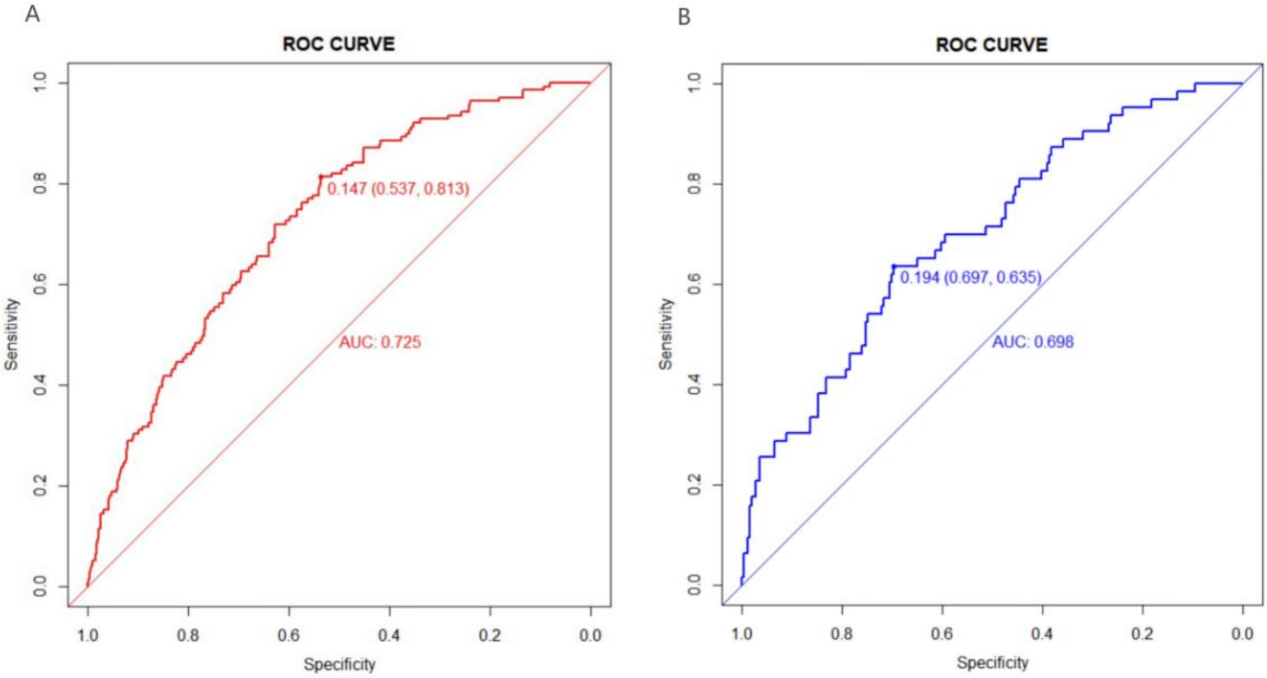

Figure 3 The ROC curves for training set (A) and validation set (B).

The part below the red line and blue line is the AUC of the model. AUC, area under curve.
