# Supplementary material for: Development and validation of a model that predicts the risk of diabetic kidney disease in type 2 diabetes mellitus patients: a retrospective study
Source: Front Endocrinol (Lausanne). 2026 Jan 13;16:1708419. doi: 10.3389/fendo.2025.1708419 (PMC12834776; doi:10.3389/fendo.2025.1708419)
Supplement: Supplementary file 5 [file Image4.pdf]

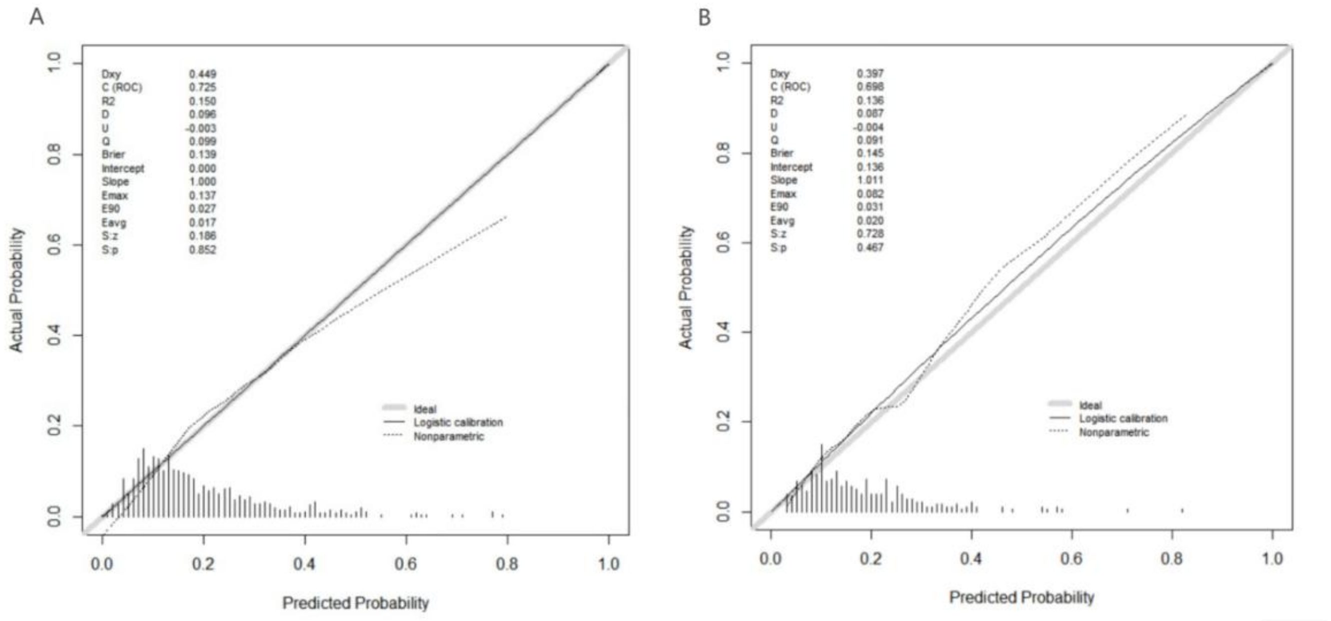

FIGURE 4 Calibration curves for training set (A) and validation set (B).

The solid line represents the model after calibration. The closer the calibration curve of the model is to the ideal line, the better the model's prediction accuracy. The DCA curves for both the training and validation sets demonstrated that this model provides good net benefits for clinically predicting the risk of DKD in patients with DKD (Figures 5A, B).
