# Supplementary material for: Development and validation of a model that predicts the risk of diabetic kidney disease in type 2 diabetes mellitus patients: a retrospective study
Source: Front Endocrinol (Lausanne). 2026 Jan 13;16:1708419. doi: 10.3389/fendo.2025.1708419 (PMC12834776; doi:10.3389/fendo.2025.1708419)
Supplement: Supplementary file 6 [file Image5.pdf]

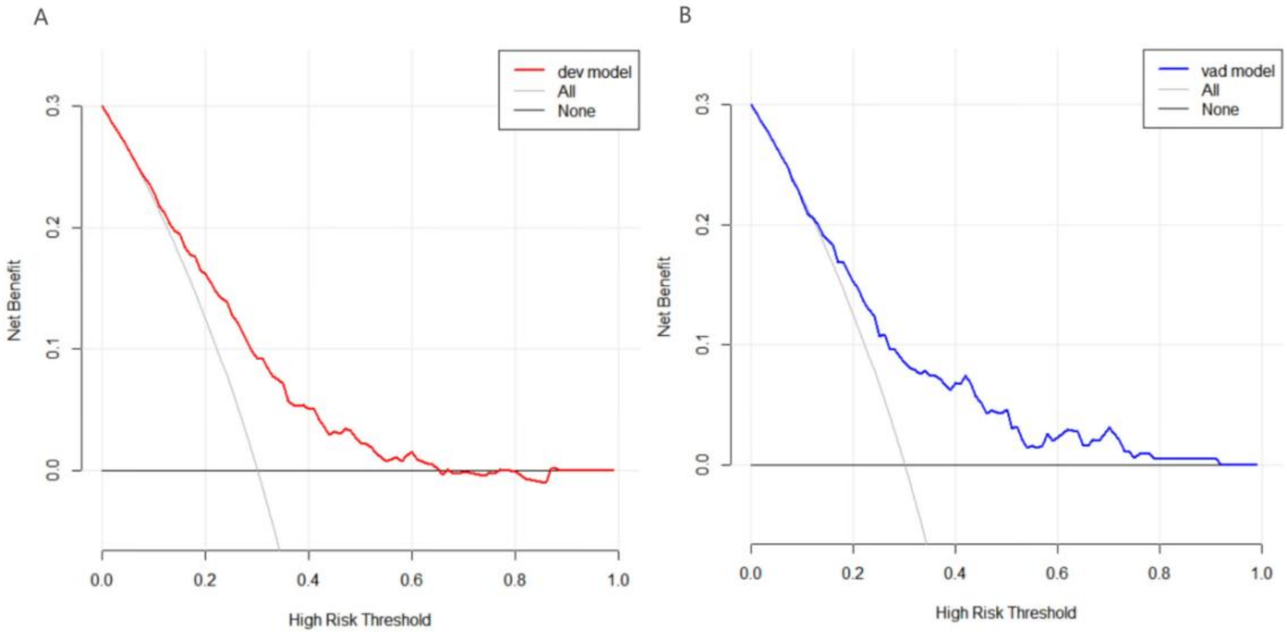

FIGURE 5 Decision curve analysis for training set (A) and validation set (B).
The dotted line indicates that all patients experienced DKD and the horizontal line represents that no patients experience DKD. The red line and blue line represents the nomogram model. The curves show that the model is clinically beneficial across a relatively wide range of threshold probabilities.
